# Biodegradable Acid-Based Fe_2_MnO_4_ Nanoparticles for Water Remediation

**DOI:** 10.3390/molecules29163867

**Published:** 2024-08-15

**Authors:** Rabia Ahmad, Elham A. Alzahrani, Poonam Dwivedi, Sumbul Hafeez, Jyoti Deswal, Bushra Fatima, Sharf Ilahi Siddiqui, Seungdae Oh

**Affiliations:** 1 Department of Chemistry, Jamia Millia Islamia, New Delhi 110025, India; rahmad@jmi.ac.in (R.A.); jyotideswal05@gmail.com (J.D.); bushrafatima89@gmail.com (B.F.); 2 Department of Chemistry, College of Science, University of Ha’il, Ha’il 81451, Saudi Arabia; elh.alzahrani@uoh.edu.sa; 3 Department of Chemistry, Ramjas College, University of Delhi, Delhi 110007, India; sharf_9793@rediff.com; 4 Department of Civil and Environmental Engineering, Villanova University, Villanova, PA 19085, USA; sumbulhafeez03@gmail.com; 5 Department of Civil Engineering, Kyung Hee University, Yongin-si 17104, Gyeonggi-do, Republic of Korea

**Keywords:** biodegradable acid, adsorption, ferrite, water remediation, dye, methylene blue

## Abstract

This study demonstrated the synthesis of Fe_2_MnO_4_ modified by citric acid, a biodegradable acid, using a simple co-precipitation method. Characterization was performed using qualitative analysis techniques such as Fourier-transformed infrared spectroscopy, scanning electron microscopy equipped with energy-dispersive X-ray spectroscopy, X-ray diffraction, selected-area electron diffraction, N_2_ adsorption–desorption, and zero-point charge. The prepared nanoparticles had a rough and porous surface, and contained oxygenous (-OH, -COOH, etc.) functional groups. The specific surface area and average pore size distribution were 83 m^2^/g and 5.17 nm, respectively. Net zero charge on the surface of the prepared nanoparticles was observed at pH 7.5. The prepared nanoparticles were used as an adsorbent to remove methylene blue dye from water under various conditions. Using small amounts of the adsorbent (2.0 g/L), even a high concentration of MB dye (60 mg/L) could be reduced by about ~58%. Exothermic, spontaneous, feasible, and monolayer adsorption was identified based on thermodynamics and isotherm analysis. Reusability testing verified the stability of the adsorbent and found that the reused adsorbent performed well for up to three thermal cycles. Comparative analysis revealed that the modified adsorbent outperformed previously reported adsorbents and unmodified Fe_2_MnO_4_ in terms of its partition coefficient and equilibrium adsorption capacity under different experimental conditions.

## 1. Introduction

Polluted water that is not fit for drinking has become a widespread problem for aquatic life [1,2]. The main reason for this problem is the wastewater generated as a result of various industrial activities [3,4,5], particularly that containing dye [6]. Dye-containing wastewater contaminates the fresh water, preventing sunlight penetration, increasing the growth of algae, and reducing the dissolved oxygen levels by increasing the chemical oxygen demand and biological oxygen demand [7].

Considering the seriousness of this problem, new technologies have been developed to maintain the purity of water [2,3,4,5,6]. Of these methods, adsorption is considered to be the most effective, least expensive, and most sustainable approach [7]. It is used to clean wastewater by removing soluble pollutants (i.e., adsorbates) using a variety of undissolved solid materials (i.e., adsorbents) [7]. The use of nanomaterials (NMs) as an adsorbent is common [8]. The advantages offered by NMs include their stability in air, cost-effectiveness, and convenient preparation in an ultra-dispersed state [8,9].

The previously reported NMs have distinct characteristics. Significant emphasis has been placed on the modification of already-reported NMs because this is a well-known process [9]. Notably, ferrites (e.g., manganese ferrite, cobalt ferrite, and copper ferrite) have been most widely used due to their advantageous morphological, magnetic, and optical properties [10,11].

Manganese ferrite (Fe_2_MnO_4_; MF) is a magnetic metal oxide nanoparticle (NP) with unique chemical and physical properties that is more magnetically susceptible than other ferrites [11]. It has a cubic spinel structure and is used in many technological applications [12,13,14]. The properties of MF depend on its size, composition, and morphology, which are all related to the preparation conditions [13]. MF can be synthesized through various methods to achieve the desired morphology and a controllable size [14]. Various MF_-_based NMs, such as a graphene oxide–MF nanocomposite (NC) [15], a magnetic polyacrylic acid–MF NC [16], a chitosan–MF NC [17], an MF/titanium oxide NC [18], a polyvinylpropyllidone-decorated MF NC [19], and an oleic acid-coated MF/polystyrene NC [20], have been reported for wastewater treatment.

According to recent studies, biomaterials have emerged as viable competitors for NMs for use in wastewater treatment [21]. Biomaterials such as biodegradable polymers [22], biomass [23], and fungi [24] have been developed as less toxic and highly functional adsorbents. However, although biomaterials are better theoretically, in practice they were initially not useful due to their non-magnetic characteristics and large particle size, thus increasing the costs of adsorption. To solve this problem, biomaterials with a higher adsorption capacity have been combined with NPs [10,11]. Prominent examples include MF– Alysicarpus Vaginalis [25], MF–neem leaves [26], and black cumin–MF–reduce graphene oxide [27], which have all proved to be excellent adsorbents. However, their low dispersibility and stability remain a problem.

In light of this, recent studies have focused on the modification of NPs through the incorporation of functional groups using biodegradable organic acids (BOCs) [28]. This type of modification leads to an improvement in the functionality and stability of NMs in water. BOCs can be degraded by many microorganisms without any environmental harm, which is an important feature [28].

Based on these characteristics, in the present study, MF was modified with the BOC citric acid (CA) using an inexpensive and facile co-precipitation method. CA is an eco-friendly and sustainable cross-linker that is widely available in nature [29]. Furthermore, CA is non-toxic, has low reactivity, and is chemically stable, and it is used as a plasticizer [30]. CA can thus soften a material and make it more flexible and less viscous [29,30]. Additionally, CA is a triprotic acid with three pKa values (3.13, 4.76, and 6.40) [31]. All of these properties are extremely important for an excellent functional adsorbent [28,29,30,31]. This is why MF is modified with CA in the present study.

In particular, this study aimed to (a) introduce functional groups from CA to MF via the modification of MF NPs using CA (CA/MF-NC) via co-precipitation, (b) investigate the physiological properties of the modified adsorbent, (c) assess the adsorption efficiency of the prepared adsorbent with methylene blue (MB), (d) conduct isotherm simulations and thermodynamic analysis, (e) confirm the reusability of the adsorbent, and (f) compare the proposed adsorbent with previously reported adsorbents.

## 2. Results and Discussion

### 2.1. Characterization

**Figure 1:**
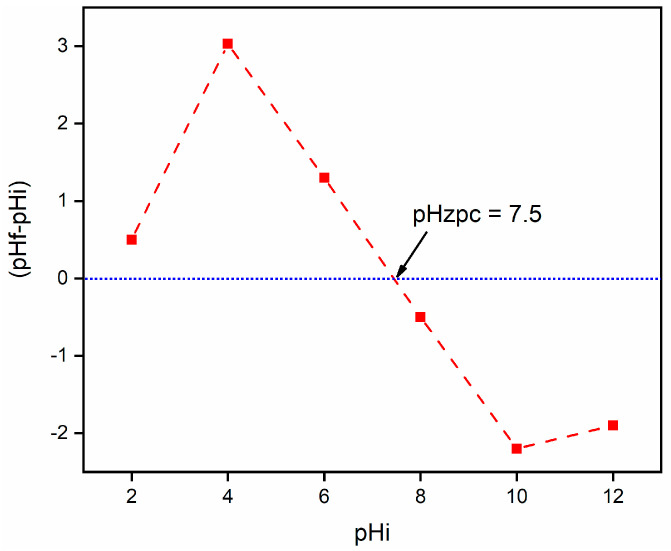
ZPC plot for CA/MF-NC.

The surface charge of CA/MF-NC was examined by measuring its ZPC values. As shown in Figure 1, the ZPC (defined as the point at which the positive charge equals the negative charge) of the composite was found to be 7.5. This suggested that, at a pH range above 7.5, the surface would acquire a negative charge (-O^−^ and -COO^−^) while, below pH 7.5, the surface would be positively charged (OH_2_^+^ and COOH_2_^+^). This can be explained by the presence of oxygenated/hydroxyl functional groups on the surface, which potentially become deprotonated (-O^−^ and -COO^−^) and protonated (OH_2_^+^ and COOH_2_^+^) with changes in the pH [32].

**Figure 2:**
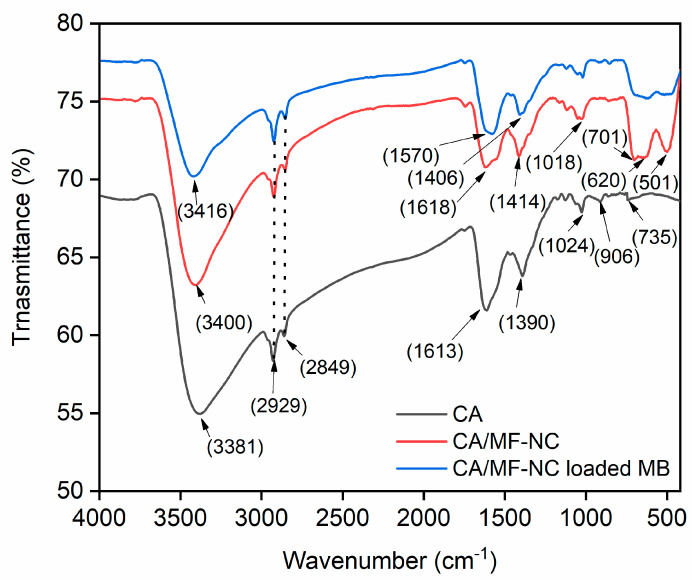
FTIR spectra for CA, CA/MF-NC before MB adsorption, and CA/MF-NC after MB adsorption.

We measured the FTIR spectrum of CA, CA/MF-NC before MB adsorption, and CA/MF-NC after MB adsorption, over the range of 400–4000 cm^−1^ to identify the functional groups and the interactions [27]. The FTIR spectrum of CA (Figure 2, black line) exhibited vibrational bands for hydroxyl and carboxylic functional groups at 3381 cm^−1^ (for O-H molecular vibrations), 1613 cm^−1^ (for carbonyl stretching), 1390 cm^−1^ (for O-H bending or C-H bending), 1024 cm^−1^ (for C-OH stretching or C-O-C vibrations), and 1000–700 cm^−1^ (for O-H bending and C-C stretching). Furthermore, the peaks at 2929 cm^−1^ and 2849 cm^−1^ confirmed that the C-H stretching mode was present in the CA.

As shown in the FTIR spectrum of CA/MF-NC (Figure 2, red line), the band at 3400 cm^−1^ in the O-H stretching mode represented the water molecules or hydroxyl groups of the NC [27]. The vibrational peaks at 1618 cm^−1^, 1414 cm^−1^, 1018 cm^−1^, and 701 cm^−1^ represented C=O stretching, O-H bending or C-H bending, C-OH or C-O-C, and C-C stretching of the carboxylic groups, respectively [25,26,27]. Furthermore, the peaks at 2929 cm^−1^ and 2849 cm^−1^ confirmed the presence of the C-H stretching mode in the CA. The stretching vibrations observed at 501 cm^−1^ and 620 cm^−1^ confirmed the presence of M-O vibration bonds, which indicated that the metal oxide NPs had integrated the functional groups of CA [27].

These bands, which were attributed to multiple functional groups, were likely present in the aggregate due to the interaction between the CA and the MF NPs [27]. However, the intensity of these peaks in CA/MF-NC was lower than those in the CA spectrum, and a slight shift in their positions was observed. The FTIR peaks that shifted in the CA/MF-NC spectrum were mainly those associated with (-OH) and (-C=O) groups. According to previous studies [27], a shift in these peaks indicates electrostatic and non-electrostatic (such as H bonding) interactions. Therefore, it can be assumed that the modification of MF in the proposed composite may have occurred through electrostatic and non-electrostatic interactions with CA.

**Scheme 1:**
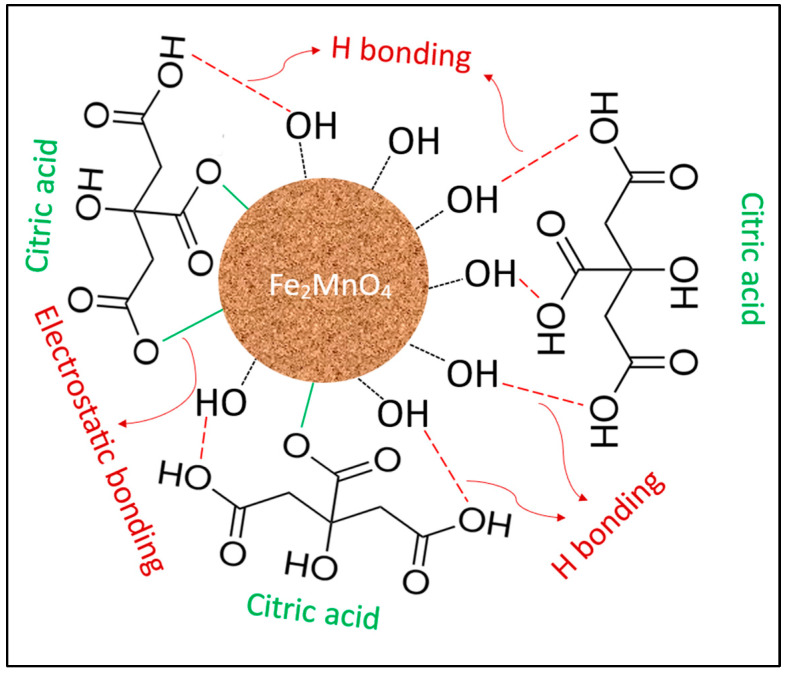
Proposed interactions between the MF NPs and the functional groups of CA.

Scheme 1 presents the proposed interactions between the -OH functional groups of MF and the functional groups of CA. The addition of FeCl_3_ and MnCl_2_, which provided Fe^3+^ and Mn^2+^ ions, to the water, in conjunction with the addition of NaOH, led to the formation of MF via co-precipitation. MF has numerous hydroxyl (-OH) functional groups in an aqueous medium (MF-OH) [13], which interacted with the functional groups of CA, including -OH and acidic (-COOH) groups, leading to the formation of CA/MF-NC (Scheme 1).

After the absorption of MB using CA/MF-NC, the FTIR spectrum of the MB-loaded composite was also analyzed. MB-loaded CA/MF-NC had similar FTIR peaks to those in CA/MF-NC with a slight shift in the wavenumber. For example, the band at 3400 cm^−1^ in the CA/MF-NC spectrum shifted to 3416 cm^−1^, while 1618 cm^−1^ shifted to 1570 cm^−1^, and 1414 cm^−1^ shifted to 1406 cm^−1^. There was no change in the peaks at 2929 cm^−1^ and 2849 cm^−1^, which indicated a specific interaction between the functional groups of CA/MF-NC and MB dye during the reaction.

**Figure 3:**
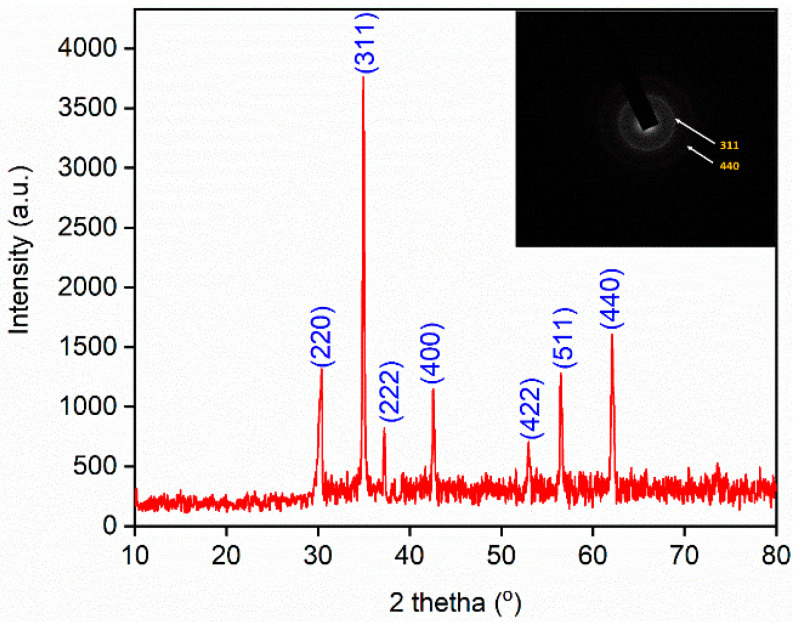
XRD pattern of the prepared CA/MF-NC (inset: SEAD pattern of CA/MF-NC).

The spinel ferrite structure and phase of the NPs were also investigated using XRD [32]. Figure 3 shows the XRD spectrum of the synthesized powdered sample, which was indexed and matched with the JCPDS Card No. 74-2403, suggesting that the phase of the sample was spinel. The peaks in the XRD pattern matched with 2*θ* values at around ~30°, ~35°, ~37°, ~43°, ~53°, ~57°, and ~62°, corresponding to the [220], [311], [222], [400], [422] [511], and [440] planes of spinel ferrite, respectively. Additional peaks that appeared may have been due to the presence of impurities [17,27]. The size of the crystallites determined from the XRD measurements using the Scherrer equation (Equation (1)) was found to be in the range of ~17.0 to 35.0 nm.

(1) Dp=(0.9×λ)/(β×Cosθ)

Here, *Dp* is the average crystallite size, *β* is line-broadening in radians (i.e., full width at half maximum), *θ* is the Bragg angle, and *λ* is the X-ray wavelength (Cu K α, *λ* = 0.154 nm).

**Figure 4:**
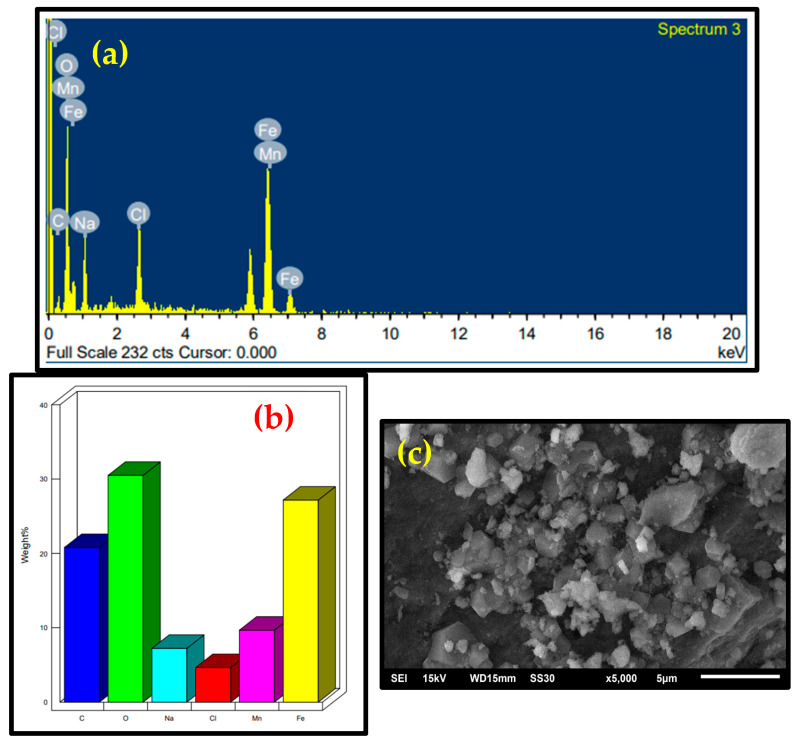
(**a**) EDAX spectrum, (**b**) quantitative analysis, and (**c**) SEM image of CA/MF-NC.

The elemental composition of the CA/MF-NC was revealed using EDX analysis at the microscopic level (Figure 4a,b). The EDX pattern clearly confirmed the presence of elements Mn, Fe, C, and O, with weight percentages of 9.67, 27.19, 20.79, and 30.51 wt.%, respectively. Na and Cl were also observed as impurities in the sample.

The surface morphology of the CA/MF-NC was investigated using SEM. An SEM micrograph of the sample at a magnification of 5000 is presented in Figure 4c, showing well-dispersed crystal-like particles with different sizes and shapes. The surface of the particles was rough, and agglomeration was observed between particles. The surface of the prepared sample was much rougher than that of pure MF, as reported in a previous study [12], which may be due to the interaction between the CA and MF.

Collectively, these results suggest that the MF NPs were modified by the interaction with CA [27,32].

**Figure 5:**
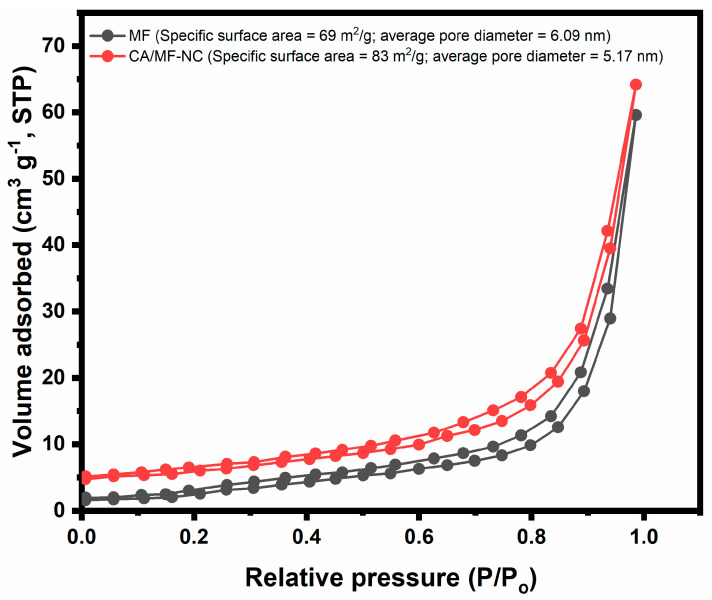
N_2_ adsorption–desorption isotherms of MF and CA/MF-NC

The specific surface area and average pore size of CA/MF-NC were measured using Brunauer–Emmett–Teller (BET) analysis. BET analysis is defined as an isotherm model that studies the adsorption of gas over a certain range of pressure. In this study, the surface area and average pore size of CA/MF-NC were measured through N_2_ gas adsorption–desorption isotherm (Figure 5). The specific surface area and average pore size of CA/MF-NC were measured to be 83.0 m^2^/g and 5.17 nm, respectively. The specific surface area of CA/MF-NC was found to be higher than that of unmodified MF, while the average pore size for modified MF was smaller than that of unmodified MF.

### 2.2. Adsorption Analysis

#### 2.2.1. Effect of the Amount of CA/MF-NC on the RE (%) for MB Dye

**Figure 6:**
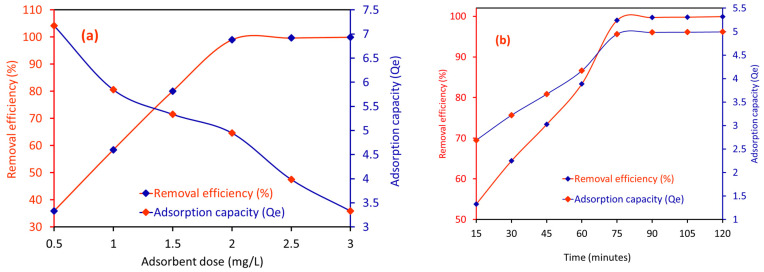

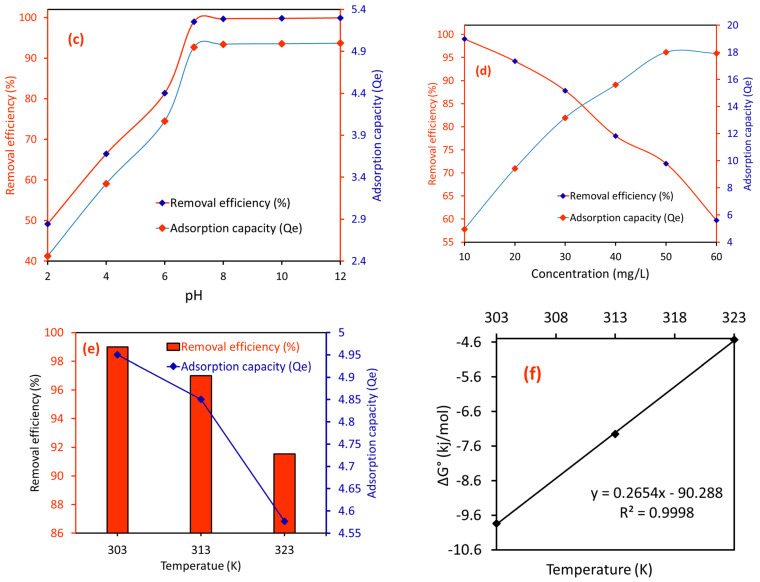
Effect of various parameters on the adsorption performance of CA/MF-NC for MB dye removal: (**a**) adsorbent dose, (**b**) contact time, (**c**) solution pH, (**d**) solution concentration, (**e**) solution temperature, and (**f**) thermodynamics.

To evaluate the effect of the dosage of CA/MF-NC on the RE (%) and adsorption capacity for MB dye, we used several 50 mL Erlenmeyer flasks containing 10 mL of 10 ppm MB solution and various amounts of CA/MF-NC (5, 10, 15, 20, 25, and 30 mg) and agitated them at 150 rpm for 2 h using a water bath shaker, before centrifuging them at 3000 rpm (Remi C-854/6, India). The results showed that, with an increase in the amount of the adsorbent from 0.5 to 3.0 g/L, the RE (%) for MB dye increased from ~36.00 to ~100.0% (Figure 6a). An equilibrium in the RE (%) was observed at 20 mg (2.0 g/L). These results were observed because, as the amount of the adsorbent increased, the number of sites available for adsorption also increased [32].

In contrast, the adsorption capacity decreased with an increase in the adsorbent dosage. This was consistent with the mathematical hypothesis of the inverse relationship between the adsorption capacity and adsorbent mass Qe=(Co−Ce)V/m. At 2.0 g/L, CA/MF-NC removed ~99.0% of 10 ppm MB dye in the water sample, so this dose was employed as the standard dose for further analysis [27].

#### 2.2.2. Effect of Time on the RE (%) of CA/MF-NC for MB Dye

To optimize the time required for the maximum removal of MB dye, several 50 mL Erlenmeyer flasks containing a fixed concentration of MB solution (10 mL at 10 ppm) and a fixed amount of CA/MF-NC (20 mg) were agitated using a water bath shaker (150 rpm) at room temperature and pH 7.0. The adsorption experiments were conducted at pH 7 because there is a balance of hydroxide (OH^−^) and hydronium (H_3_O^+^) or hydrogen (H^+^) ions at this pH. In acidic or alkaline solutions, dyes can compete with H^+^ or OH^−^ ions to find active sites on the adsorbent surface.

To analyze the effect of time, the agitated sample was centrifuged every 15 min and the concentration of the centrifuged aqueous samples was analyzed using a UV–Vis spectrophotometer. The RE (%) of CA/MF-NC for MB was found to increase from 56.83 to 99.23% with an increase in the contact time from 15 to 75 min, respectively (Figure 6b). The adsorption capacity also increased with an increase in the contact time, from ~2.70 to ~5.0 mg/g between 15 and 75 min. The removal rate was more rapid at the beginning of the contact time and slowed down as the contact time increased [25]. This may be because, at the start, all of the adsorbent sites were empty, and the concentration gradient of the solute was steep. As the contact time increased, it gradually approached equilibrium at 75 min.

#### 2.2.3. Effect of pH on the RE (%) of CA/MF-NC for MB Dye

The charge on the adsorbent surface is controlled by the pH of the solution, which affects how the adsorbent and adsorbate interact. In particular, the RE of an adsorbent can differ depending on the pH. It is thus important to determine the optimal pH range for an absorbent because the pH of natural water can change with the addition of pollutants. To analyze the effect of pH on the performance of CA/MF-NC as an adsorbent, several 50 mL Erlenmeyer flasks containing a fixed concentration of MB solution (10 mL at 10 ppm) and a fixed amount of CA/MF-NC (20 mg) were adjusted to a pH range of 2–12 using 0.1 N HCl and 0.1 M NaOH and agitated using a water bath shaker (150 rpm) at room temperature for 2 h. The solutions were then centrifuged at 3000 rpm and the MD concentrations at various pH levels was analyzed using a UV–Vis spectrophotometer. The CA/MF-NC surface charge was influenced by the pH (ZPC = 7.5, which represents a slight negative charge) of the dye solution. The cationic MB dye molecules are positively charged; thus, a lower pH did not favor the adsorption of MB onto CA/MF-NC, while a higher pH did promote adsorption (Figure 6c). This is because, at an alkaline pH, the negatively charged NC becomes more negatively charged due to deprotonation, thus attracting cationic MB ions and promoting adsorption [27].

#### 2.2.4. Effect of MB Concentration on the RE (%) of CA/MF-NC for MB Dye

To investigate the effect of the dye concentration (10–60 ppm) on the MB removal rate using 20 mg of CA/MF-NC, we agitated a set of 50 mL flasks using a water bath shaker at 150 rpm and room temperature for 2 h, after which the solution mixtures were centrifuged at 3000 rpm. The concentrations of the centrifuged aqueous samples were then analyzed using a UV–Vis spectrophotometer. The results indicated that 2.0 g/L CA/MF-NC was able to eliminate more than 60% of the MB from a solution with an initial MB concentration of 60 ppm. As the initial concentration increased from 10 ppm to 60 ppm, the elimination efficiency (%) of CA/MF-NC for MB gradually decreased (Figure 6d), which might be due to the limited adsorptive sites on the fixed amount of CA/MF-NC. However, the reduction in the RE (%) for CA/MF-NC was not very high, suggesting that the high efficiency of CA/MF-NC for the removal of MB might be due to the large number of surface sites on CA/MF-NC [27].

In contrast, the adsorption capacity increased with an increase in the initial concentration of the MB solution. This was consistent with the mathematical hypothesis of the proportional relationship between the adsorption capacity and the initial MB concentration: Qe=(Co−Ce)V/m.

#### 2.2.5. Effect of Temperature on the RE (%) of CA/MF-NC for MB Dye and Associated Thermodynamics

We studied the influence of the reaction temperature at 30 °C (303 K), 40 °C (313 K), and 50 °C (323 K) using an initial MB concentration of 10 ppm and a 20 mg dosage of CA/MF-NC. The influence of the reaction temperature on the RE (%) and adsorption capacity is shown in Figure 6e, which indicates that the MB uptake from the solution decreased with an increase in the temperature. This trend illustrated the exothermic nature of MB adsorption [27]. The temperature-dependent adsorption data were also employed to calculate the thermodynamic variables, including the enthalpy change (Δ*H*°), Gibbs free energy change (Δ*G*°), and entropy change (Δ*S*°). These parameters can be used to determine the heat change during the reaction and the feasibility and spontaneity of the adsorption reaction. These variables were calculated utilizing the following equations (Equations (2) and (3)) [27]:

(2) $$\;\Delta G^{o}=\Delta H^{o}-Τ\Delta S^{o}$$

(3) $$\Delta G^{o}=-RΤlnKc$$

where *Kc* (equilibrium constant) = *Qe*/*Ce*.

The results obtained from these equations are summarized in Table 1. The negative values for ∆*G*° (−9.838, −7.251, and −4.529 kJ/mol at 303 K, 313 K, and 323 K, respectively) indicate that the process was thermodynamically feasible and spontaneous (Figure 6f). The Δ*G*° for a spontaneous process range between 0 and −20 kJ/mol, indicating the physisorption type of the MB adsorption process [25,27]. ∆*H*° was calculated to be −90.288 kJ/mol, which represents exothermic behavior with a probability of favorable adsorption [25,27]. The entropy value of −0.265 kJ/mol/K indicated the affinity of MB towards CA/MF-NC. It suggests a decrease in the system’s degrees of freedom, showing that MB is fixed onto the surface of CA/MF-NC [25,27].

#### 2.2.6. Adsorption Isotherm Analysis

##### Langmuir Isotherm

The Langmuir adsorption isotherm assumes a uniform and homogeneous adsorption process with monolayer coverage, implying that all adsorption sites exhibit consistent and equivalent surface coverage. A molecule’s capacity to adsorb at one location is unaffected by the occupancy of other sites. The Langmuir model considers the sites’ linear form, assumes that they are equivalent [27], and can be written as Equation (4):

(4) $$\frac{C_{e}}{Q_{e}}=\frac{C_{e}}{Q_{o}}+\frac{1}{Q_{o}b}$$

where *C_e_* is the equilibrium concentration of MB, *Q_e_* is the equilibrium adsorption capacity, *Q_o_* is the maximum adsorption capacity of the adsorbent in a monolayer, and b represents the Langmuir constant, which is associated with the adsorption energy. Additionally, the separation factor (*R_L_*) is determined as follows (Equation (5)) [27]:

(5) $$R_{L}=\frac{1}{\left(1+bC_{e}\right)}$$

where *C_e_* is the equilibrium concentration of MB and *R_L_* represents the possible adsorption method. For *R_L_* > 1, the adsorption method is considered unfavorable, if *R_L_* = 0, the adsorption method is irreversible, and for 0 < *R_L_* < 1, the adsorption method is considered energetically favorable.

##### Freundlich Isotherm

This confirms that the surface of CA/MF-NC is not uniform and that each adsorption site has a distinct bond energy. The Freundlich isotherm parameters can be determined by the following equation (Equation (6)) [27]:

(6) $$logQ_{e}=logK_{F}+\frac{1}{n}logC_{e}$$

where *C_e_* is the equilibrium concentration of MB, *Q_e_* is the equilibrium adsorption capacity, and *K_F_* [(mg g^−1^) (L mg^−1^)^1/*n*^] and *n* are the Freundlich constants that indicate the adsorbent’s capacity for solute molecules and the surface heterogeneity. The Langmuir and Freundlich factors are summarized in Table 2.

**Figure 7:**
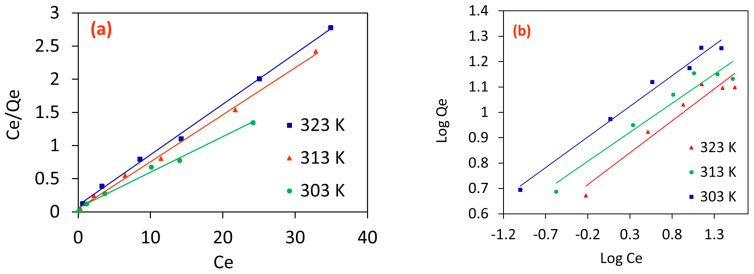
Results for the (**a**) Langmuir and (**b**) Freundlich linear isotherms

These parameters suggest that the Langmuir model offers a better fit than the Freundlich model, with the correlation coefficients (R^2^) for the Langmuir model being close to 1 for all tested temperatures (Figure 7a,b). Therefore, this suggests that the MB molecules form a monolayer on the surface of CA/MF-NC. The decrease in *Q*_max_ with temperature indicates the exothermic nature of MB adsorption onto the surface of CA/MF-NC. The values of *R_L_* were found to be in the range 0 < *R_L_* < 1, suggesting that the adsorption method was energetically favorable.

#### 2.2.7. Reusability Testing

The quality of an adsorbent depends on its reusability and stability. In the present study, thermal therapy was used to examine the regeneration and reusability of CA/MF-NC. In this process, 1.0 g of CA/MF-NC loaded with MB was rinsed with double-distilled water and subsequently dried at 60 °C in a hot-air oven for 24 h. The resulting adsorbent was heated at 100 °C, 200 °C, 300 °C, or 400 °C in a muffle furnace [27,33]. By heating CA/MF-NC loaded with MB at high temperatures, the MB is pyrolyzed, thus leaving the surface of the absorbent in gaseous form. After pyrolysis, the carbon content remaining on the surface of the adsorbent was removed by washing it several times with double-distilled water [27,33]. The regenerated adsorbent was then dried and reused for the next adsorption cycle. Regeneration and reuse were investigated for up to five cycles.

The results of the present study found that the RE (%) of reused CA/MF-NC initially increased with an increase in the temperature due to the higher regeneration caused by the pyrolysis of MB at higher temperatures (Table 3). However, when the temperature increased further (above 200 °C), it was found that the RE (%) of reused CA/MF-NC decreased. This may be because CA decomposes at temperatures above 200 °C, causing most of the surface functional groups to disappear, and this leads to a decrease in the functional group-driven RE (%) of CA/MF-NC.

It was also found that, as the number of reuse cycles increased, the RE (%) decreased. However, this decrease was not significant for the first three cycles.

#### 2.2.8. Performance Evaluation and Comparative Analysis

It is not appropriate to directly compare the MB adsorption capacity and RE (%) of CA/MF-NC with other adsorbents reported in previous studies because previous adsorbents have been used under a range of different adsorbent doses, reaction temperatures, solution pH levels and concentrations, contact times, and agitation rates. Thus, the partition coefficient (PC) was employed in the present study to assess the performance of these adsorbents and allow for a comparative analysis. The use of the PC eliminates bias in the comparative analysis of two adsorbents tested under different conditions. The equation for the PC is as follows (Equations (7) and (8)):

(7) PC=Adsorption capacity (Qe)/Final concentration (Ce)

(8) PC=Adsorption capacity (Qe)/(Initial concentration (Co)×removal rate (Qt))

Table 4 summarizes the calculated PC, equilibrium adsorption capacity (*Qt*), and RE (%) for previously reported adsorbents and CA/MF-NC under the various experimental conditions. The PC for CA/MF-NC was 64.9 L/g, which was higher than those of the other adsorbents. CA/MF-NC had a higher *Qt* (5.0 mg/g) and RE (99.2) at a lower adsorbent dose (2.0 g/L) than the other adsorbents. The PC, *Qt*, and RE (%) of the CA/MF-NC were also compared with those of pristine MF NPs, whose PC, *Qt*, and RE (%) were much lower than those of CA/MF-NC, thus confirming that CA modification improves the adsorption performance of MF.

## 3. Chemicals and Preparation

### 3.1. Chemicals

CA was purchased from a local market in Jamia Millia Islamia (New Delhi-110025, India). Salts of manganese and iron (MnCl_2_, purity: 99.99%; FeCl_3_, purity: 97.0%), MB dye (C_16_H_18_C_l_N_3_S, purity: 97.0%), NaOH (purity: 97.0%), and HCl (purity: 37.0%) were procured from Sigma-Aldrich Corporation, Bangalore, India. All chemicals were used as purchased without any further purification.

### 3.2. Synthesis of CA/MF-NC

This study reports the novel modification of MF NPs with CA. A simple co-precipitation method reported in an earlier study [12] was used to modify MF NPs with CA to obtain CA/MF-NC. In brief, to synthesize CA/MF-NC, aqueous solutions of a 0.1 M ferric chloride solution and a 0.05 M manganese chloride solution (100 mL each) were mixed under continuous stirring at 50 °C. After stirring the resulting mixture for another 20 min, 100 mL of a 0.05 M CA solution was added under continuous stirring. The resulting mixture was stirred for another 40 min at 50 °C. Then, an 8 M NaOH solution was added dropwise to the solution mixture until the pH of the solution became 11–12. This reaction resulted in the formation of a brown precipitate, which was centrifuged at 3000 rpm (Remi C-854/6, Delhi, India). The precipitate was washed first with distilled water and then with excess double-distilled water to remove the unreacted CA and other residues (i.e., Cl and Na ions). The resulting product was used for characterization and absorption analyses.

### 3.3. Development of the MB Stock Solution

MB is a cationic and carcinogenic dye that was used as the model pollutant for the present study. It has an absorption maximum at 660 nm and a molar mass of 319.85 g/mol. To prepare a 1000 PPM stock solution of MB, 1000 mg of the MB was dissolved in 1000 mL of distilled water and diluted to 10 ppm, 20 ppm, 30 ppm, 40 ppm, 50 ppm, and 60 ppm using the dilution formula N_1_V_1_ = N_2_V_2_ [32].

### 3.4. Instrumentation and Characterization

The characterization of the prepared CA/MF-NC was carried out using various spectroscopic and microscopic techniques, including Fourier-transformed infrared spectroscopy (FTIR) [27,28], scanning electron microscopy (SEM) [27] equipped with energy-dispersive X-ray spectroscopy (EDS) [32], X-ray diffraction (XRD) [27], selected-area electron diffraction (SAED) [39], and Brunauer–Emmett–Teller (BET) [27]. The FTIR results for CA/MF-NC were recorded on a Perkin Elmer spectrophotometer (Waltham, MA, USA). An XRD analysis of CA/MF-NC powder was conducted using a Model No. XtaLAB Synergy Make Rigaku, Japan. SEM-EDX analysis was conducted using a Model No. JSM 6510LV Make JEOL, Japan. SAED analysis was performed with F30 S-Twin, Technai, Eindhoven, The Netherlands. The BET-specific surface area and average pore size distribution of CA/MF-NC were measured using a Tristar 3000 BET surface analyzer (Micromeritics, Norcross, GA, USA).

To determine whether the surface charge of the adsorbent was positive or negative, the zero-point charge (ZPC) was assessed. To achieve this, a ZPC experiment was conducted using the salt addition method with a 0.1 M KNO_3_ solution [32]. Briefly, 20 mL of the 0.1 M KNO_3_ solution was added to several flasks. The initial pH (pHi) of each solution was adjusted to a range of 2–12 by adding 0.1 M NaOH and 0.1 N HCl. Following this, 20 mg of CA/MF-NC was added to each solution, and the mixture was shaken using a water bath shaker for 4 h at room temperature and 150 rpm. After the 4 h of shaking, the mixtures were equilibrated for 12 h, and then the final pH (pHf) of the equilibrated mixture was recorded. A plot of the differences in the pH (pHf-pHi) was plotted against pHi and the point where the two curves intersected determined the ZPC. The pH of each solution before and after agitation was recorded in triplicate with a calibrated pH meter (METTLER TOLEDO, Mumbai, India).

### 3.5. Adsorption Analysis

Adsorption experiments for the removal of MB dye using the prepared CA/MF-NC adsorbent were performed in a batch system. These experiments were carried out with 10 mL of a dye solution in a series of 50 mL Erlenmeyer flasks. These flasks were shaken using a water bath shaker at 190 rpm for various time intervals under different reaction conditions. In the present study, the removal of MB dye from the aqueous solutions was measured in terms of the removal efficiency (RE; %) and adsorption capacity (*Qe*) at varying adsorbent doses (0.5–3.0 g/L), initial MB concentrations (10–60 mg/L), solution temperatures (303–323 K), contact times (15–120 min), and pH levels (2–10). RE (%) and *Qe* were determined using Equations (9) and (10), respectively:

(9) RE (%)=(Co−Ce/Co)×100

(10) Qe (mg/g)=(Co−Ce)V/m

where *m* (g/L) is the mass of CA/MF-NC used, *V* is the volume of the MB solution (10 mL), *Co* (mg/L) is the initial MB concentration in the solution before adsorption, *Ce* (mg/L) is the final MB concentration in the solution after adsorption, and *Qe* (mg/g) is the adsorption capacity of CA/MF-NC at equilibrium. The initial and the final concentrations of the MB dye in the solution in the Erlenmeyer flasks were measured spectrophotometrically using a UV–visible spectrophotometer (T80-UV/VIS), PG Instruments Limited, Leicestershire, UK, at 660 nm. Details of the experimental conditions are provided in the relevant Section 2. All of the experiments were conducted in triplicate and the mean of the results was reported. The obtained results were used for further thermodynamic and isothermal analysis.

## 4. Conclusions

In this research, the co-precipitation method was implemented for the synthesis of biodegradable CA-modified MF NPs. The successful preparation and physicochemical properties of the modified NPs were confirmed using various techniques. An SEM with EDX analysis revealed the synthesis of rough and crystal-like MF NPs modified by CA (CA/MF-NC). This was supported by XRD patterns confirming their spinel structure. The specific surface area of the modified NPs was found to be higher compared to that of the unmodified NPs. The FTIR spectrum of the prepared NPs exhibited vibrational peaks for functional groups due to CA being adsorbed onto the MF. The pH-ZPC of the prepared modified NPs was observed at pH 7.5. The adsorptive removal of MB dye using CA/MF-NC as an adsorbent was also tested. The negative values for the Gibbs free energy, enthalpy, and entropy suggested that the MB adsorption process was thermodynamically feasible, unconstrained, and exothermic. The adsorption rate was also better described by the Langmuir isotherm model than the Freundlich model. Comparative analysis showed that the modified MF NPs showed a better performance in removing MB dye from water compared with previously reported adsorbents and unmodified MF NPs.

## Figures and Tables

**Table 1:** Thermodynamics results for MB adsorption.

Order	Temperature, (K)	∆*G*°	∆*H*°	∆*S*°
1.	303	−9.838	−90.288	−0.265
2.	313	−7.251		
3.	323	−4.529		

**Table 2:** Results of the isotherm models.

Order	Temperature, (K)	Langmuir Isotherm				Freundlich Isotherm		
		*Q*_max_, (mg/g)	*β* (L/mg)	*R_L_*	R^2^	*K_F_* (mg/g) (L/mg)^1/*n*^	N	R^2^
1.	303	18.587	0.895	0.100	0.992	9.939	4.135	0.985
2.	313	14.124	1.407	0.066	0.997	7.133	4.370	0.935
3.	323	13.140	0.746	0.118	0.998	5.830	3.976	0.933

**Table 3:** Results of reusability testing.

Order	Temperature, °C	Reuse Cycle				
		First	Second	Third	Forth	Fifth
1.	100	84	73	62	56	43
2.	200	90	77	66	59	48
3.	300	86	71	61	54	47
4.	400	78	64	56	50	43

**Table 4:** Comparative analysis of the proposed CA/MF-NC adsorbent and those from previous studies. Adapted from Tara et al. [27].

Order	Adsorbent	Adsorbent Dose (g/L)	Contact Time (min)	Solution pH	Temperature (°C)	Initial Concentration (mg/L)	Equilibrium Adsorption Capacity (mg/g)	Removal Efficiency (%)	PC (L/g)	Ref.
1.	MnFe_2_O_4_/BC	3.0	45.0	7.0	27.0	10.0	3.3	99.4	52.6	[32]
2.	Ag-Ag_2_O/ZrO_2_/GL	2.0	30.0	7.0	27.0	10.0	4.9	99.0	50.6	[34]
3.	CuO	2.5	30.0	6.0	22.0	25.0	5.5	54.7	0.5	[35]
4.	*Haloxylon recurvum* plant stems	4.0	40.0	8.0	25.0	20.0	4.8	96.4	6.7	[36]
5.	*Solanum tuberosum* plant leaves	2.0	24.0	7.0	30.0	10.0	3.9	79.0	1.9	[37]
6.	Fe_2_O_3_-SnO_2_/BC	2.0	90.0	7.0	27.0	10.0	4.9	97.9	23.9	[38]
7.	MF NPs	2.0	75.0	7.0	27.0	10.0	2.8	55.5	20.7	Present study
8.	CA/MF-NC	2.0	75.0	7.0	27.0	10.0	5.0	99.2	64.9	

## Data Availability

Data are contained within the article.
